# New Perspectives on Analyzing and Interpreting Base Running Efficiency: A GPS Approach

**DOI:** 10.3390/s26082378

**Published:** 2026-04-12

**Authors:** José Antonio Martínez-Rodríguez, Jonathon Neville, John B. Cronin

**Affiliations:** 1 Sport Research Institute New Zealand, Auckland University of Technology, Auckland 1010, New Zealand; jono.neville@aut.ac.nz (J.N.); john.cronin@aut.ac.nz (J.B.C.); 2 Athlete Training and Health, Houston, TX 77494, USA

**Keywords:** monitoring, technology, baseball, curvilinear running, sprinting

## Abstract

Base running performance in baseball depends on the ability to efficiently transition between linear and curvilinear sprinting; however, current assessment approaches provide limited insight into how speed is developed, maintained, or lost across these phases. This perspective presents a methodological framework for using GPS technology to enhance the analysis and interpretation of base running performance through segment-specific velocity and time diagnostics. GPS data were collected during 54.7 m linear sprints and home-to-second-base curvilinear sprints in three high-school baseball players with differing performance profiles. Sprint paths were divided into standardized linear (L1–L4) and curvilinear (C1–C4) segments, allowing examination of speed changes between successive phases to identify acceleration, maintenance, and deceleration patterns. Comparative case analyses illustrate how athletes differ in their ability to negotiate the curve around first base, reaccelerate toward second base, and maintain speed under increasing curvilinear demands. In addition, a base running efficiency ratio (BREr) is introduced to quantify how effectively linear sprint capacity is preserved during curvilinear base running, both globally and across early and late phases of the sprint. The three players’ data illustrated that GPS-derived velocity–time profiles may provide useful insights into individual running strategies, path selection, and segment-specific performance limitations that are not captured by traditional timing methods. Rather than establishing normative benchmarks, this paper emphasizes the applied value of GPS technology as a diagnostic tool to potentially inform individualized assessment and monitoring in applied settings related to linear and curvilinear sprint performance in baseball.

## 1. Introduction

Teams with good hitters will allow runners on base, and those runners will create opportunities to take extra bases and score runs. The ability to run between and around bases, therefore, will impact offensive performance and team success in baseball, highlighting the critical importance of both linear and curvilinear sprint abilities [1,2,3]. Given that base running involves both linear and curvilinear sprinting characteristics, it is important to understand if these motor qualities have distinct technical and physical demands [1,4]. Martinez-Rodriguez, Crotin, Neville and Cronin [1] investigated the influence of linear and base running speed measures on stealing a base (linear) and running two bases (curvilinear) from Baseball Savant, Baseball Reference, and Lahman databases from 475 Major League Baseball players. The authors reported that a linear increase across the speed, acceleration, and base running speed metrics quartiles was observed from players who stole the least (0–25%) to the most (75–100%) bases. In contrast, no linear increase in the metrics was observed across the quartiles from players who ran the least (0–25%) to the most (75–100%) doubles (running two bases). It would seem those players who were fast in a straight line (running single bases) were different to those running curvilinear paths (running multiple bases).

Although base running-specific sprinting remains under-researched, 200 m sprinters who run curves present with an inward body lean, asymmetrical force application, and different functional roles for the inside and outside foot to maintain speed while redirecting the center of mass [5,6,7,8,9,10,11,12]. Compared with straight sprinting, curvilinear sprinting is characterized by reduced anteroposterior force, increased mediolateral force, and altered lower-limb kinematics, with the inside foot contributing more to redirection and stabilization and the outside foot to forward propulsion of the center of mass [6,7,8,9]. Researchers studying the electromyography of elite sprinters have reported specific muscle activation of the gluteus medius and bicep femoris in the outside leg, and the semitendinosus and adductor muscles in the inside leg. Specifically, the vastus lateralis and tibialis anterior were activated, while the gastrocnemius medialis was highly activated in both legs during the curvilinear sprint segment [12,13]. This supports the notion that curvilinear sprinting is a distinct neuromechanical task rather than simply linear sprinting performed on a curved path [12,13,14]. Although base running differs from a lane-constrained sprint compared to a 200 m track event, base runners similarly need to maintain speed while negotiating the curve around the bases. Therefore, evidence from curvilinear track events provides an important theoretical framework for understanding the technical demands of baseball-specific curvilinear sprinting and transfer of capacities between linear and curvilinear sprints [4,15,16].

Given these differences between linear and curvilinear sprinting and inside–outside foot mechanics, it was concluded that there was a need to implement base running assessments to understand linear and curvilinear sprinting base running ability. Current methods for evaluating base running typically rely on timing gates or stopwatch technology, which limit the depth of understanding of spatiotemporal and kinematic characteristics across the curvilinear sprint [1,17,18]. These timing technologies are unable to capture critical aspects that might indicate a lack of technique, such as speed loss during the curvilinear segment, and do not map individual approaches and self-selected curvilinear paths when running multiple bases. Therefore, other diagnostic methods are needed that provide detailed information about the time and speed during linear and curvilinear sprinting. Global positioning system (GPS) devices are one such technology that can potentially provide more granular information about in situ base running. In this paper, the utility of GPS technology as a tool for the implementation, collection, and interpretation of segment-specific velocity and time metrics for diagnostic base running performance is explored. Using representative athlete case-study examples, the analysis illustrates how GPS-derived data can be used to examine differences between linear single-base sprinting and curvilinear multi-base running. The purpose of this work is therefore to present a practical analytical framework for interpreting GPS-derived base-running metrics, rather than to establish generalized performance principles.

## 2. Collecting Base Running Data Using GPS Technology

**Figure 1:**
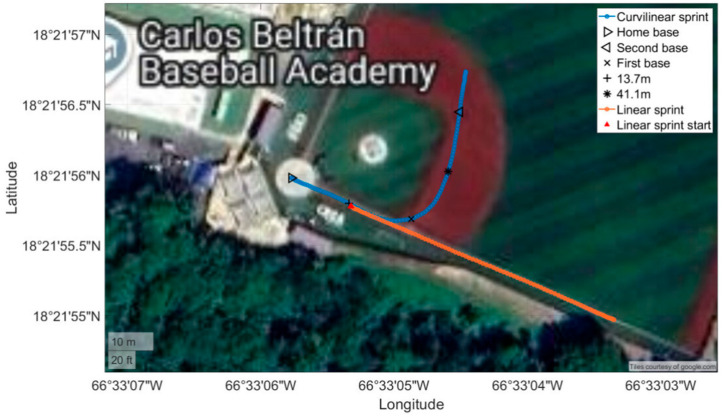
Samples collected during the straight-line and curvilinear sprints.

GPS technology determines the position of a receiver using trilateration, whereby the receiver estimates its location based on measured distances from multiple satellites. To resolve the three-dimensional position (X, Y, and Z axes) of the unit and the receiver clock bias, signals from at least four satellites are required. The GPS device used sampled positional and velocity data at 10 Hz using an eighth-generation Swiss high-precision chipset (VX Sport, Wellington, New Zealand). The unit supports multiple satellite constellations, including GPS, GLONASS, QZSS, SBAS, Galileo, and BeiDou, allowing for improved satellite visibility and positioning robustness during outdoor athletic performance monitoring. The device operates as a multi-GNSS receiver but does not utilize real-time kinematic (RTK), precise point positioning (PPP), or differential GNSS correction methods. The unit includes a nine-axis inertial measurement unit (IMU) composed of three-axis accelerometers, gyroscopes, and magnetometers, each sampled at 100 Hz, allowing detailed monitoring of body motion. The GPS device was affixed posteriorly between the scapulae in a vest to maintain antenna orientation and minimize movement. Approximately 30 min prior to data collection, calibration coordinates were recorded for home, first and second base locations along with the start and end of the 54.7 m sprint (see Figure 1). The unit was then powered off to save the calibration data in a separate file, and subsequently powered on again to enable satellite signal recognition and stabilization approximately 15 min before each testing session [17].

Both linear and curvilinear (home to second base) sprint tests were recorded over 54.7 m (collected in the same session), with linear sprint ability assessed first. Each session began with a standardized dynamic warm up focused on lower body movements. Two trials for each test were performed, separated by three minutes of rest between trials and ten minutes of rest between tests. The home-to-second-base sprint starting position was a stealing-base lead-off position at home plate with the front foot on top of the home plate. The participant would then sprint, rounding first base, before reaching and passing through second base. The linear sprint starting position was a stealing-base lead-off position at home plate with the front foot on top of the middle of the starting line. Therefore, the linear test was complementary to the curvilinear test, as the linear sprint reflects the raw capacity to sprint and the curvilinear sprint demonstrates the body’s technical and physical curve negotiation, from which base running efficiency ratios can be calculated.

All trials were conducted in baseball-specific shoes on a turf surface, and athletes were instructed to run maximally without slowing down before reaching the endpoint. After each data collection, the data was uploaded and stored in the cloud for later analysis. GPS curvilinear data was processed using a custom data-processing algorithm [19].

Understanding the core variables for each segment allows for calculation of the peak before first base and peak after first base. The speed data between the home plate and the first base reference location was used to identify the highest recorded speed, which was defined as the peak speed before first base (see Figure 1). Next, within the first and second bases, the search was iterated to determine the highest recorded speed after the first base and before the second base. To capture any change in speed that occurred between these two peaks in speed, the data was examined to identify the lowest recorded speed, defined as the minimum speed between the two bases.

The reliability of the GPS-derived measures used in the following sections was previously established by Martínez-Rodríguez et al. Martínez-Rodríguez, et al. [20]. The authors evaluated 12 male high school baseball players, who completed three testing sessions separated by at least 48 h. In each testing session players performed 54.7 m linear and curvilinear base-running (home-to-second-base) sprints. The authors evaluated multiple performance variables derived from the GPS velocity–time profile, including split times, peak sprint velocities, minimum velocities during curve negotiation, and segment-specific running speeds and distances at standardized points along the running path (e.g., 13.7 m, 27.4 m, and 41.1 m). Reliability was quantified using intraclass correlation coefficients (ICC) to assess relative reliability and coefficients of variation (CV%) to assess absolute reliability across the three testing sessions. Acceptable between-session reliability for most of the GPS-derived variables was reported, with coefficients of variation ranging from 1.3–7.7% for linear sprint measures and 1.6–8.4% for curvilinear base-running variables. Intraclass correlation coefficients for most measures were reported as moderate to high (ICC~0.74–0.98), indicating consistent ranking of participants across testing sessions. Additionally, the authors calculated the smallest worthwhile change (SWC) to determine the sensitivity of the GPS-derived metrics for detecting meaningful performance changes. The analysis showed that several key metrics, particularly time and velocity measures at distances of 41.1 m and 54.7 m, as well as running speed at 27.4 m, were adequately sensitive to detect performance changes. Collectively, these findings indicated that the GPS-based measurement system provided reliable and consistent estimates of time-, speed-, and distance-based variables during both linear and curvilinear baseball sprinting tasks. It would seem that GPS might be appropriate for applied performance monitoring and diagnostic analysis in baseball athletes

The next three sections will focus on the analysis and interpretation of the data processed from the GPS device, specifically focusing on the curvilinear and linear differences between segments (i.e., the time in the current segment minus the time in the previous segment). These differences provide insight into how base runners change their speed across successive running segments.

- A positive difference reflects an increase in speed, indicating the base runner accelerated into the next segment.
- A negative difference reflects a decrease in speed, indicating the base runner decelerated into the next segment.
- A difference close to zero suggests the base runner maintained similar speed between segments.

This approach allows identification of where speed is gained, maintained, or lost throughout the sprint and provides a clearer understanding of segmental performance.

## 3. Analysing and Interpreting Linear Base Running Ability

Three high school baseball players (Player A, Player B, and Player C) from a baseball academy in Puerto Rico were selected for in-depth performance comparison. The players varied in time to second base, which allowed the demonstration of the utility of the GPS data analysis across different performance profiles. This approach illustrates how baseball coaches and sport scientists can apply the comparative framework to their baseball players, enabling individualized monitoring.

**Figure 2:**
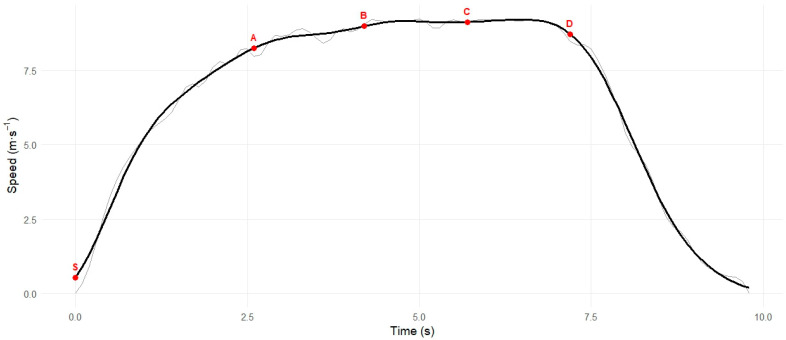
Speed–time profiles of a baseball player performing a 54.7 m linear sprint. Key: S = Start; A = Time and speed at 13.7 m; B = Time and speed at 27.4 m; C = Time and speed at 41.1 m; D = Time and speed at 54.7 m.

As can be observed in Table 1, the distance was divided into four linear sprint (L) segments (see Figure 2), and the velocities for each linear distance quantified via GPS:

- L1 (0–13.7 m)—indicative of initial acceleration
- L2 (13.7–27.4 m)—indicative of transitional acceleration to maximum speed
- L3 (27.4–41.1 m)—indicative of maximal speed
- L4 (41.1–54.7 m)—indicative of speed endurance

Linear sprint data is reported in Table 1, the differences in speed development among Players A, B, and C highlighted. Player A had the fastest performance overall, maintaining higher speeds consistently between segments. Player C decelerated early from L2 to L3, indicating poor transitional acceleration. The three players decelerated before passing the 54.7 m mark.

To evaluate how each player transitions between bases, calculating the difference between segments provides a clear indication of whether the base runner is increasing or decreasing speed across these segments. Specifically:

- L2–L1—This phase reflects how much speed changes when moving from initial acceleration to transitional acceleration.
- L3–L2—This phase reflects the transition from acceleration toward maximal speed.
- L4–L3—This phase reflects the player’s ability to maintain maximal speed during the later portion of the sprint.

The linear sprint speed changes across successive segments (L1, 2, 3, and 4) are presented in Table 2, and three observations can be made from these data:

- L2–L1: Player A demonstrated the largest increase in speed (0.80 m·s^−1^) during this phase as compared to Players B and C, indicating greater speed development during the transition from initial acceleration.
- L3–L2: There was very little increase in speed during this phase (−0.19 to 1.0 m·s^−1^), indicating that players were approaching maximal speed; however, most notable was that Player C was already decelerating during this phase.
- L4–L3: Speed decreased during this phase in all three players, indicating a reduction in speed during the later stages of the sprint, with the largest decrease observed in Player A (−0.40 m·s^−1^).

From a practical perspective, these linear segment-specific velocity profiles illustrate how practitioners might interpret GPS-derived sprint data to identify potential areas of performance development. Specifically:

1. The speed reduction observed in the later phase of the sprint for Player A may suggest a potential focus on speed endurance capacity.
2. The relatively smaller speed increases during the early sprint phases for Players B and C may indicate areas where initial or transitional acceleration development could be considered.
3. The reduction in speed observed for Player C during the L3–L2 phase illustrates how practitioners might examine maximal speed development or speed maintenance characteristics when interpreting segment-specific sprint profiles.

## 4. Analyzing and Interpreting Curvilinear Base Running Ability

**Figure 3:**
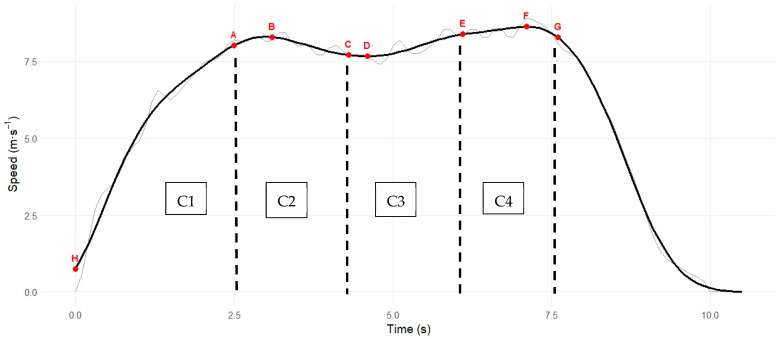
Speed–time profiles of a baseball player performing a home-to-second-base sprint. Key: H = Home; A = Time at 13.7 m; B = Peak speed before first base; C = Time and speed at first base; D = Minimum speed between bases; E = Time at 41.1 m; F = Peak speed before second base and after first base; G = Time and speed at second Base; C1 = Curvilinear segment 1; C2 = Curvilinear segment 2; C3 = Curvilinear segment 3; C4 = Curvilinear segment 4.

As can be observed in Table 3, the distance was divided into four curvilinear (C) segments (Figure 3), and the speed for each curvilinear distance quantified via GPS:

- C1 (Home plate—13.7 m)—indicative of initial acceleration.
- C2 (13.7 m—first base)—indicative of curvilinear deceleration.
- C3 (First base—41.1 m)—indicative of curvilinear reacceleration.
- C4 (41.1 m—second base)—indicative of maximal speed.

Curvilinear sprint data is reported in Table 3, the differences in speed, time and distance among Players A, B, and C highlighted. Player A demonstrated the best overall performance relative to Players B and C. From C1 to peak speed, Player A exhibited the smallest increase in velocity (+0.17 m·s^−1^) compared with Player B (+0.37 m·s^−1^) and Player C (+0.34 m·s^−1^). After reaching peak speed, Player A also was found to have substantially lower deceleration toward first base (−0.45 m·s^−1^) than Player B (−1.15 m·s^−1^) and Player C (−1.56 m·s^−1^), suggesting less deceleration during the transition. After C2, Player A again maintained speed more effectively, with lower deceleration (−0.61 m·s^−1^) than Player B (−1.21 m·s^−1^) and Player C (−1.78 m·s^−1^). In contrast, Player C displayed the greatest reacceleration after peak speed (+1.59 m·s^−1^), indicating greater reliance on linear sprint ability compared with Player A (+0.79 m·s^−1^) and Player B (+1.07 m·s^−1^). All players decelerated before reaching second base.

In terms of distance covered across the four segments, distinct curvilinear path strategies were observed between players. In C1 (0–13.7 m), Player C covered the greatest distance (13.32 m), followed by Player A (13.15 m) and Player B (12.54 m), indicating a slightly longer initial path for Player C. During C2 (13.7 m to first base), Player C again covered the greatest distance (14.66 m), while Player B (14.20 m) and Player A (13.88 m) followed, suggesting a wider approach into first base for Player C. In contrast, Player A covered the greatest distance in C3 (first base to 41.1 m: 14.97 m) compared with Player B (14.43 m) and Player C (13.22 m), indicating a more progressive curvilinear trajectory between bases. During the final segment (C4), Player C (13.48 m) and Player B (13.42 m) covered greater distances than Player A (12.51 m), suggesting that Player A adopted a tighter running line when approaching second base. When comparing Player A with Players B and C, Player A demonstrated a wider running path in C3 exiting the first base, whereas Players B and C consistently adopted wider running paths in C2 approaching first base.

To evaluate how each player transitions between sprint segments, calculating the difference between segments provides a clear indication of whether the base runner is increasing or decreasing speed.

- C2–C1—This phase reflects the change in speed when the base runner enters the curve approaching first base.
- C3–C2—This phase reflects the change in speed associated with reacceleration after negotiating the curve.
- C4–C3—This phase reflects the base runner’s ability to accelerate to maximum speed or maintain speed when exiting the curve toward second base.

The curvilinear sprint speed changes across successive segments (C1, 2, 3, and 4) are shown in Table 4, and three points are important to highlight:

- C2–C1: Player A demonstrated the smallest reduction in speed (−0.28 m·s^−1^) during the entry into the curve and covered the shortest distance during this phase as compared to Players B and C. This may suggest a tighter running path with less disruption to speed. In contrast, Players B and C appeared to adopt a different strategy where they covered more distance with a larger reduction in speed to set themselves up with a different exit path/strategy around first base.
- C3–C2: Player A was observed to have minimal speed change (−0.05 m·s^−1^), indicating stable speed in this phase. Players B and Player C demonstrated greater increases in velocity during this phase, suggesting a more pronounced reacceleration after first base. Player C also exhibited a slightly flatter curve (i.e., −1.44 m).
- C4–C3: Players B and C exhibited substantial reductions in speed, whereas Player A ran a shorter distance, probably due to the wider path taken in the previous phase. Differences in segment distance across this phase may reflect variations in running trajectory following the curve.

From a practical perspective, these curvilinear segment-specific speed profiles illustrate how practitioners might interpret GPS-derived sprint data to identify potential areas of performance development. Specifically:

1. The minimal speed developed observed during the curve phase for Player A may suggest opportunities to examine physical/technical strategies after negotiating the curve.
2. The speed patterns observed in the later segment for Player B illustrate how practitioners might focus on reacceleration capacity when exiting the curve toward second base.
3. The trajectory and velocity changes observed for Player C across segments may warrant emphasis on curved-running mechanics and path selection.

## 5. Base Running Efficiency Ratio—Curvilinear/Linear Diagnostics

Of interest is how a base runner’s raw linear ability translates to curvilinear base running performance, revealing the extent to which linear speed potential is preserved or lost when sprinting a curved path. This potential can be calculated as a base running efficiency metric derived from the ratio of curvilinear to linear sprint in each of the phases. Base running efficiency ratio (BREr) provides insight into how a baseball player maintains their maximal linear sprint ability while rounding the bases [4]. To determine BREr, the base runners performed a linear sprint covering a distance equivalent to the 54.7 m home-to-second-base sprint. BREr is calculated by dividing the curvilinear sprint segment time over each segment by their linear sprint segment time:

BREr = (Curvilinear segment time)/(Linear segment time)

This ratio normalizes the result to each athlete’s base running ability. A value closer to 1.00 indicates that the base runners maintained a similar performance to their straight sprint performance, reflecting high efficiency (maneuverability) relative to their linear capacity (continuous acceleration) [4,15,21]. In contrast, a value farther from 1.00 suggests a substantial loss in performance during the curvilinear segment (drop in speed), reflecting low efficiency relative to their linear capacity (deceleration instead of maintenance). The BREr reflects how efficient athletes transfer linear sprinting characteristics into the curvilinear technical and physical demands of base running. It captures not only raw speed but also highlights physical and technique fluctuations when rounding bases. Therefore, a BREr of 1.05 means the runner maintains 95% of their straight-line sprint time, indicating a 5% reduction in time during curvilinear sprinting.

Base running efficiency has typically been expressed as a ratio of total time of both the linear and curvilinear 54.7 m sprints [4,16]. When computing and understanding BREr, time is the best measure to use, as neither distance nor speed are good representations of efficiency on their own; taking a wider line could increase speed, or decreasing speed could flatten the line. It therefore makes sense to look at time to each specific point as the best measure of efficiency, as it incorporates both distance and speed. However, there is a need to distinguish between global efficiency (the fastest possible time to second base vs. linear) and segmented performance. At the global level, efficiency is unambiguous, being the run that reaches the end point with the least time lost compared to linear sprinting. When broken into quarters (for any variable) it is no longer valid to assume that the smallest difference in time, distance or speed between linear and curvilinear represents better efficiency. Practitioners could fall into the trap where a participant can appear “inefficient” in one quarter (larger linear–curvilinear gap) yet still be globally efficient if that strategy reduces the gap in later quarters. It may be more prudent to break efficiency down into an early (home to first), late (first to second) phase, and total (home to second) phase because it may offer greater ecological validity. These phases closely reflect the principal base running demands encountered in baseball game situations and may therefore be easier for practitioners to interpret and apply. However, further research is needed to determine optimal segmentation strategies. This does not necessarily indicate what is most efficient, but optimal efficiency may reflect a balance of efficiency in these zones (i.e., base running efficiency ratio closer to 1.00 in each zone), which is potentially easier to understand than a complex balancing act between four split segments.

As can be observed in Table 5, the home-to-second-base sprint was divided into three BREr segments, and the ratio for each segment was quantified via BREr calculation ([curvilinear segment time]/[linear segment time]):

- BREr1 from home to first base—indicative of efficiency in maintaining speed under increasing curvilinear demands
- BREr2 from first to second base—indicative of efficiency after curvilinear demands to maximal speed.
- BREr3 from home to second base—indicative of how effective the athlete is across multiple bases and is a combination of BREr1 and BREr2.

Given the information in Table 5, several interpretations can be drawn. Player A demonstrated the highest efficiency across all three ratios, suggesting a strong ability to maintain speed given the curvilinear demands of running multiple bases. The highest BREr was observed in the first-to-second-base segment. This segment may represent a potentially relevant area for further exploration in a practice context. For example, practitioners might consider examining inside–outside foot mechanics or related strength qualities, or exploring alternative approach paths in the preceding segment, to determine whether such adjustments influence efficiency in the subsequent phase. However, these considerations should be interpreted as exploratory and context-dependent rather than prescriptive.

Limitations. Several limitations should be acknowledged when interpreting the present findings. First, the detailed comparative analysis presented in this case study is based on three high school baseball players, and therefore the results should be interpreted as illustrative examples of the analytical framework rather than general performance characteristics. Second, the participants were drawn from a specific population of high school players, which may limit the applicability of these observations to other competitive levels or sports contexts, and should be interpreted with this in mind. Finally, although GPS-derived velocity and trajectory data provide valuable information about running dynamics, the technology does not directly measure biomechanical variables such as joint kinematics, force production, or ground reaction forces [22]. Consequently, interpretations regarding movement strategies should be considered indirect inferences based on velocity–time data rather than direct biomechanical measurements.

## 6. Conclusions

GPS-derived velocity–time variables can provide a useful framework for examining the dynamics of base running performance during both linear and curvilinear sprinting. Specifically, the framework illustrates how GPS-derived data can be used to examine where athletes accelerate, maintain velocity, or experience reductions in speed while transitioning from linear sprinting into curved running around the bases. This segmented approach allows practitioners to visualize how velocity changes across different phases of the base running path and to identify potential differences in how athletes negotiate the transition from a linear to curvilinear movement.

GPS-derived metrics can reveal individual differences in segment-specific running characteristics. These characteristics include initial acceleration, transitional velocity changes approaching first base, velocity reduction during curve negotiation, subsequent reacceleration toward second base, and deceleration in the late phase associated with fatigue-related constraints on force production and stride mechanics [23]. These examples illustrate how the velocity–time profile might be interpreted to examine different base running strategies across athletes. The BREr provides an example of how relative performance between linear and curvilinear sprinting segments may be quantified to assess how effectively athletes maintain linear sprint capability while negotiating the technical demands associated with rounding bases. Therefore, the use of GPS to enhance base running ability diagnostics represents a novel method to advance understanding of linear–curvilinear interaction in base running, and potentially could better inform individualized assessment and training decisions.

## Figures and Tables

**Table 1:** Linear sprint segments: time and speed for three players different in performance.

	Player A	Player B	Player C
L1: Speed at 13.7 m (m·s^−1^)	8.22	6.96	5.95
L1: Time at 13.7 m (s)	2.60	2.95	3.30
L2: Speed at 27.4 m (m·s^−1^)	9.02	7.39	6.32
L2: Time at 27.4 m (s)	4.20	4.90	5.50
L3: Speed at 41.1 m (m·s^−1^)	9.12	7.45	6.13
L3: Time at 41.1 m (s)	5.70	6.70	7.70
L4: Speed at 54.7 m (m·s^−1^)	8.72	7.13	5.95
L4: Time at 54.7 m (s)	7.20	8.55	9.90

**Table 2:** Linear segments: change comparison across three players.

	Player A	Player B	Player C
L2–L1 (m·s^−1^)	0.80	0.43	0.37
L3–L2 (m·s^−1^)	0.10	0.06	−0.19
L4–L3 (m·s^−1^)	−0.40	−0.32	−0.18

**Table 3:** Curvilinear sprint segments: time, speed and distance for three players.

	Player A	Player B	Player C
C1: Speed at 13.7 m (m·s^−1^)	8.14	6.38	5.58
C1: Time at 13.7 m (s)	2.45	2.90	3.20
Peak speed before first base (m·s^−1^)	8.31	6.75	5.92
C2: Speed at first base (m·s^−1^)	7.86	5.61	4.36
C2: Time at first base (s)	4.15	5.15	5.85
Minimum speed between peaks (m·s^−1^)	7.70	5.54	4.14
C3: Speed at 41.1 m (m·s^−1^)	8.09	6.46	5.79
C3: Time at 41.1 m (s)	6.05	7.45	8.65
Peak speed after first base and before second base (m·s^−1^)	8.49	6.61	5.73
C4: Speed at second base (m·s^−1^)	8.08	5.00	4.65
C4: Time at second base (s)	7.55	9.60	11.10
C1: Distance covered from 0–13.7 m (m)	13.15	12.54	13.32
C2: Distance covered from 13.7 m to first base (m)	13.88	14.20	14.66
C3: Distance covered from first base to 41.1 m (m)	14.97	14.43	13.22
C4: Distance covered from 41.1 m to second base (m)	12.51	13.42	13.48

**Table 4:** Differences in speed and distance between successive curvilinear segments (C1–C4) during the home-to-second-base sprint.

	Player A	Player B	Player C
Speed (m·s^−1^)			
C2–C1	−0.28	−0.77	−1.22
C3–C2	0.23	0.85	1.43
C4–C3	−0.01	−1.46	−1.14
Distance (m)			
C2–C1	0.73	1.66	1.34
C3–C2	1.09	0.23	−1.44
C4–C3	−2.46	−1.01	0.26

Key: Negative values indicate that the preceding segment had a greater value than the subsequent segment, reflecting reductions in speed or distance between phases.

**Table 5:** BREr for three players of different curvilinear performance.

	Player A	Player B	Player C
BREr1	0.99	1.05	1.06
BREr2	1.13	1.22	1.19
BREr3	1.05	1.12	1.12

## Data Availability

The data presented in this study are available on request from the corresponding author due to privacy.

## Abbreviations

The following abbreviations are used in this manuscript:

BREr	Base running efficiency ratio
CV	Coefficient of variation
